# Relationships between intra-pancreatic fat deposition and lifestyle factors: a cross-sectional study

**DOI:** 10.3389/fendo.2023.1219579

**Published:** 2023-07-27

**Authors:** Kento Mitsushio, Megu Y. Baden, Sarasa Kato, Akiko Niki, Harutoshi Ozawa, Saori Motoda, Chisaki Ishibashi, Yoshiya Hosokawa, Yukari Fujita, Ayumi Tokunaga, Takao Nammo, Junji Kozawa, Iichiro Shimomura

**Affiliations:** ^1^ Department of Metabolic Medicine, Graduate School of Medicine, Osaka University, Suita, Japan; ^2^ Department of Lifestyle Medicine, Graduate School of Medicine, Osaka University, Suita, Japan; ^3^ Department of Diabetes Care Medicine, Graduate School of Medicine, Osaka University, Suita, Japan

**Keywords:** lifestyle factors, ectopic fat, fast eating, intra-pancreatic fat deposition, late-night eating

## Abstract

**Aims:**

The excess deposition of intra-pancreatic fat deposition (IPFD) has been reported to be associated with type 2 diabetes, chronic pancreatitis, and pancreatic ductal adenocarcinoma. In the current study, we aimed to identify a relationship between lifestyle factors and IPFD.

**Materials and methods:**

99 patients admitted to the Osaka University Hospital who had undergone abdominal computed tomography were selected. We evaluated the mean computed tomography values of the pancreas and spleen and then calculated IPFD score. Multiple regression analyses were used to assess the associations between IPFD score and lifestyle factors.

**Results:**

Fast eating speed, late-night eating, and early morning awakening were significantly associated with a high IPFD score after adjusting for age, sex, diabetes status and Body Mass Index (p=0.04, 0.01, 0.01, respectively).

**Conclusion:**

The current study has elucidated the significant associations of fast eating speed, late-night eating, and early morning awakening with IPFD.

## 1 Introduction

With the significant surge in the prevalence of obesity, organ dysfunction linked to ectopic fat accumulation is receiving a lot of attention in these days. Ectopic fat accumulation in the pancreas is termed “intra-pancreatic fat deposition” (IPFD) (1). A small amount of intra-pancreatic fat is a constituent of the normal human pancreas (1) and the amount of intra-pancreatic fat increases with age and more common in people of Asian descent (1). The excess of IPFD, which is termed “fatty pancreas disease (FPD)”, has been significantly associated with type 2 diabetes mellitus (2, 3), chronic pancreatitis (4), and pancreatic ductal adenocarcinoma (5).

Several previous studies have reported an association between lifestyle factors and IPFD (1). A randomized controlled trial showed that the use of the Mediterranean diet resulted in a significantly lower IPFD (6). In patients with post-pancreatitis, it was also reported that smoking and alcohol consumption may be associated with IPFD (7).

To elucidate whether IPFD is associated with specific dietary habits and other lifestyle factors, we previously reviewed medical records from the Osaka University Hospital, and reported that the consumption of two meals per day was associated with IPFD in patients with type 2 diabetes mellitus (8). In the current study, we conducted a prospective survey to assess more detailed lifestyle factors through the use of multiple questionnaires and examined the association between lifestyle factors and IPFD.

## 2 Methods

### 2.1 Study design and participants

The participants were patients who were admitted in the Department of Metabolic Medicine, Osaka University Hospital, between June 2021 and August 2022, and had undergone abdominal computed tomography (CT) scans for underlying diseases during their hospital stay or within 3 months before admission. The sample size was set according to our previous research (8). We excluded patients with type 1 diabetes mellitus, pancreatic diseases (e.g., pancreatic tumors or pancreatitis), endocrine diseases (e.g., Cushing syndrome or pheochromocytoma), or taking oral corticosteroid (prednisolone 10 mg or more). Diagnosis of pancreatic cancer and pancreatitis was made by experienced radiologists. The study was approved by the institutional ethics review board of Osaka University Hospital (approval number: 21062). The participants were informed about the study and gave written consent to participate.

### 2.2 Assessment of lifestyle factors

We collected information on lifestyle factors through self-administered questionnaires distributed to the patients during the hospitalization period. For dietary habits, a validated food frequency questionnaire developed by the Japan Public Health Center (JPHC) (9, 10), including 128 food items and 13 beverage items, was used. For exercise habits, the International Physical Activity Questionnaire short form (11) was used to yield a score in MET-hours/week. The participants were also asked about the number of meals consumed per day, snacking habits, timing of each meal, and eating speed. Information about eating speed and sleeping habits (sleep duration, frequency of difficulty falling asleep, mid-awakening, early morning awakening and deep sleep disorder) were collected using a questionnaire based on the JPHC-NEXT interview form (12). The questionnaire on these lifestyle factors is shown in **Supporting Information Table S1**. Smoking were assessed by the Brinkman index (number of cigarettes consumed per day multiplied by years of smoking) (13). Late-night eating was defined as eating after 9:00 p.m., and fast eating was defined as those who responded that their eating speed was fast/very fast according to previous study (14). Short sleep duration was defined as sleeping 5 hours or less per day (15).

### 2.3 Measurement of ectopic fat accumulation in the pancreas and liver

In order to measure the extent of IPFD and intra-hepatic lipid accumulation (IHLA), unenhanced CT values were utilized, which have been demonstrated to exhibit strong correlation with histologically determined organ fat content (16). As previously reported (8, 17), we determined the CT values of the pancreas (P) by calculating the mean value of three 1cm^2^ regions of pancreas (head, body, and tail). The measurements were carefully conducted by excluding the pancreatic duct and margins from the selected areas. Likewise, the CT values of the liver (L) was determined by calculating the mean CT value of three 1cm^2^ regions of liver (anterior, posterior, and lateral). The CT values of the spleen (S) was determined by calculating the mean CT value of three 1cm^2^ regions of spleen (upper, middle, and lower). Subsequently, by following previous studies, we quantified IPFD by calculating the difference between the mean pancreatic values and the mean splenic values (P-S) (8, 17, 18). Similarly, we quantified IHLA by calculating the difference between the mean liver values and the mean splenic values (L-S) (17, 19). Lower CT values indicate higher levels of fat accumulation. Measurements were performed by an experienced physician blinded to each patient’s information. To determine inter-observer variability in IPFD and IHLA measurements, another physician randomly selected 27 patients and independently measured their IPFD and IHLA. The intraclass correlation coefficients (ICC) and Bland-Altman plot between these two physicians were evaluated for these measurements. All unenhanced CT scanning was performed with a slice thickness of 5 mm. The images were analyzed using Aquarius Net Viewer Version 4.4 (TeraRecon, Inc., Tokyo, Japan).

### 2.4 Covariate assessment

The following data was obtained from the medical records at the time of participants’ admission: age; sex; height; body weight; Hemoglobin A1c (HbA1c); fasting plasma glucose; triglycerides (TG); high-density lipoprotein-cholesterol (HDL-C); and low-density lipoprotein-cholesterol (LDL-C) concentrations. Blood samples were obtained in the morning, prior to breakfast, on the day following admission. Diabetes mellitus were diagnosed according to Japanese diagnostic criteria (20).

### 2.5 Statistical analysis

The P-S and L-S values were square root transformed to normalize their distributions and then multiplicated to the range of 0–100 (with 0 representing lowest fat deposition and 100 as highest fat deposition). The numbers were defined as “IPFD score” and “IHLA score”, respectively. Multiple regression analyses were conducted to assess the associations between each lifestyle factor and the scores of IPFD and IHLA, adjusting for covariates including age (continuous), sex (male or female), diabetes status (presence of diabetes mellitus), and body mass index (BMI, continuous). Least squares geometric mean concentrations were calculated for lifestyle factors. The results were showed as Model 1, not adjusted; Model 2, adjusted for age and sex; Model 3, adjusted for age, sex, and diabetes status; and Model 4, adjusted for age, sex, diabetes status, and body mass index. In addition, to evaluate generalizability, sensitivity analysis was performed by stratifying by the presence or absence of diabetes. Each macro-nutrient and micro-nutrient intake provided by FFQ is adjusted for total energy intake by residual method. Statistical analyses were performed using R version 4.2.2. (https://www.r-project.org/) and two-sided p-value < 0.05 was considered to represent statistical significance. Any missing data were excluded from the statistical analyses.

## 3 Results

### 3.1 Participant characteristics

**Figure 1:**
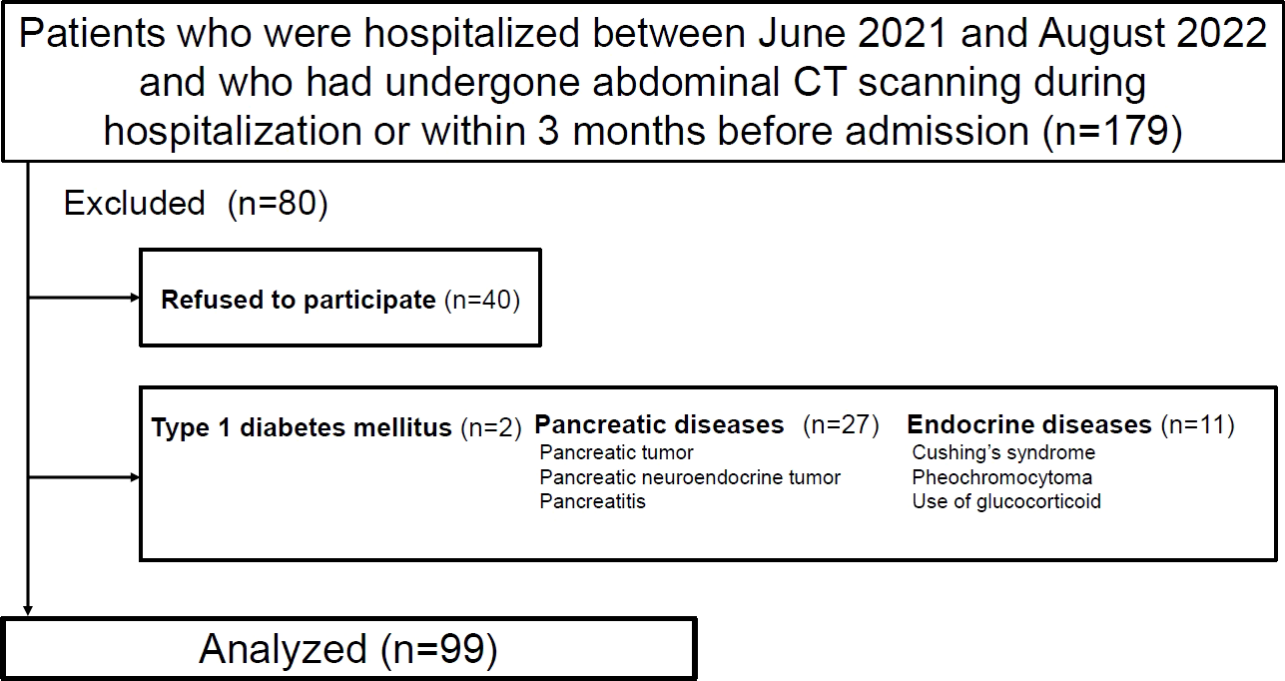
Flow chart of the recruitment of participants.

Ultimately, 99 patients were enrolled in the study (**Figure 1**). **Table 1** presents the clinical characteristics of the participants upon hospitalization. The median age was 64 years and median BMI was 25.3 kg/m^2^. The median CT values for the pancreas, liver and spleen were 40.0 HU, 56.9 HU and 49.0 HU, respectively. ICC (2,1) for IPFD (P-S) was 0.86 (95%CI: 0.71-0.93) and ICC (2,1) for IHLA (L-S) was 0.98 (95%CI: 0.96-0.99). Bland-Altman plots for IPFD and IHLA were shown in **Supporting Information Figures S1**, **2**. For both measurements, more than 95% values were included within the limits of agreement. No significant fixed errors (p = 0.08 and 0.41, respectively) and proportional errors (p = 0.59 and 0.39, respectively) were observed. IPFD score 18.0 and IHLA score 18.7 correspond to 1SD.

**Table 1:** Clinical characteristics of the participants.

	n=99
Age (years)	64.0 (50.5, 72.5)
Sex (male/female)	52/47
Dietary habits	
Number of meals/day (two/three/four)	15/82/2
Number of snacks/day (none/one/two/three)	21/47/28/3
Time of last meal	19:00 (18:45, 20:00)
Eating speed (very slow/slow/mid/fast/very fast) (n=98)	6/2/36/45/9
Alcohol intake (g/day) (n=97)	0.0 (0.0, 2.0)
Exercise habits	
Physical activity (MET-hour/week)	16.5 (4.5, 38.2)
Smoking habits	
Brinkman Index (n=93)	100 (0, 400)
Sleeping habits	
Sleep duration (hours/day)	6.0 (6.0, 7.0)
Difficulty falling asleep (days/week)	2.0 (0.0, 3.0)
Mid-awakening (days/week)	3.0 (2.0, 7.0)
Early morning awakening (days/week)	0.0 (0.0, 2.0)
Deep sleep disorder (days/week)	2.0 (0.5, 3.0)
Mean CT values for the pancreas (HU)	40.0 (31.7, 45.0)
Mean CT values for the liver (HU)	56.9 (49.8, 61.4)
Mean CT values for the spleen (HU)	49.0 (45.9, 51.6)
Intra-Pancreatic Fat Deposition score (IPFD score)	48.2 (39.1, 61.4)
Intra-Hepatic Lipid Accumulation score (IHLA score)	48.6 (42.2, 60.6)
Visceral fat area (cm^2^) (n=97)	126.6 (84.8, 164.2)
Subcutaneous fat area (cm^2^) (n=97)	170.7 (111.3, 228.7)
Body mass index (kg/m^2^)	25.3 (22.1, 29.1)
Type 2 diabetes mellitus (patients)	68
HbA1c (NGSP, %) (n=96)	7.5 (5.8, 8.8)
Fasting plasma glucose (mmol/L) (n=95)	6.4 (5.1, 8.1)
HDL-C (mmol/L) (n=96)	1.2 (1.0, 1.5)
LDL-C (mmol/L) (n=96)	2.8 (2.2, 3.4)
TG (mmol/L) (n=98)	1.3 (0.9, 1.9)
Reasons for admission	
Diabetes management (patients)	57
Adrenal function evaluation (patients)	22
Improvement of obesity (patients)	4
Pituitary function evaluation (patients)	2
Primary hyperparathyroidism evaluation (patients)	2
Anemia workup (patients)	2
Thyroid cancer treatment (patients)	2
Others (patients)	8
Reasons for CT	
Preoperative evaluation (patients)	22
Adrenal evaluation (patients)	21
Malignant tumor screening (patients)	16
Malignant tumor assessment (patients)	8
Pancreas evaluation (patients)	6
Liver evaluation (patients)	5
Postoperative evaluation (patients)	5
Abdominal pain workup (patients)	3
Anemia workup (patients)	2
Others (patients)	11

Values are median (first quartile, third quartile) for continuous variables and number of participants for categorical variables. NGSP, National Glycohemoglobin Standardization Program. HDL-C, high-density lipoprotein cholesterol; LDL-C, low-density lipoprotein cholesterol; TG, triglycerides; CT, Computed tomography.

### 3.2 Relationship between lifestyle factors and IPFD

We examined the associations of IPFD score with lifestyle factors (**Table 2**) and nutrient intakes (**Supporting Information Table S2**). Fast eating speed, late-night eating, low alcohol intake, early morning awakening and deep sleep disorder were significantly associated with high IPFD score after adjusting for age and sex (p=0.02, 0.01, 0.03, 0.02 and 0.03, respectively). After further adjustment for diabetes status and BMI, fast eating speed, late-night eating, and early morning awakening were significantly associated with high IPFD score (p=0.04, 0.01 and 0.01, respectively). For nutrient intakes, vitamin B12 intake tended to be negatively associated with IPFD in the age, sex, diabetes, and BMI adjusted model (p=0.05).

Table 2Relationship between lifestyle factors and IPFD score.

**Table:** 

Eating habits		
Two meals/day (Yes/No)	Coefficient (95% CI)	p-value
Model 1	0.5 (-9.5, 10.5)	0.92
Model 2	3.9 (-6.1, 14.0)	0.45
Model 3	6.0 (-4.1, 16.0)	0.25
Model 4	4.0 (-6.0, 14.1)	0.43
Number of snacks (per once/day increase)		
Model 1	1.2 (-3.4, 5.9)	0.61
Model 2	1.2 (-3.7, 6.0)	0.64
Model 3	0.3 (-4.7, 5.2)	0.92
Model 4	-0.2 (-5.1, 4.6)	0.93
Fast eating (Yes/No)		
Model 1	6.7 (-0.5, 13.9)	0.07
Model 2	8.2 (1.2, 15.2)	0.02
Model 3	7.1 (-0.03, 14.2)	0.05
Model 4	7.5 (0.6, 14.4)	0.04
Late-night eating after 9:00 p.m. (Yes/No)		
Model 1	8.6 (-1.5, 18.8)	0.10
Model 2	12.9 (2.7, 23.1)	0.01
Model 3	13.1 (3.1, 23.2)	0.01
Model 4	12.6 (2.7, 22.4)	0.01
Alcohol intake (per 1g/day increase)		
Model 1	-0.2 (-0.4, -0.004)	0.05
Model 2	-0.2 (-0.4, -0.02)	0.03
Model 3	-0.2 (-0.4, 0.01)	0.06
Model 4	-0.2 (-0.4, 0.03)	0.10

**Table:** 

Exercise habits		
Physical activity (per 1MET-hours/week increase)		
Model 1	0.005 (-0.1,0.1)	0.91
Model 2	0.02 (-0.1, 0.1)	0.68
Model 3	0.03 (-0.05, 0.1)	0.42
Model 4	0.03 (-0.1, 0.1)	0.49

**Table:** 

Smoking		
Brinkman index (per 1 increase)		
Model 1	0.01 (-0.002, 0.02)	0.13
Model 2	0.005 (-0.004, 0.01)	0.27
Model 3	0.004 (-0.004, 0.01)	0.34
Model 4	0.004 (-0.004,0.01)	0.38

**Table:** 

Sleeping habits		
Short sleep duration (Yes/No)		
Model 1	7.1 (-1.6, 21.6)	0.11
Model 2	7.5 (-1.0, 16.0)	0.09
Model 3	8.9 (0.4, 17.3)	0.04
Model 4	7.3 (-1.1, 15.8)	0.09
Difficulty falling asleep (per 1day/week increase)		
Model 1	0.1 (-1.4, 1.6)	0.94
Model 2	0.1 (-1.3, 1.6)	0.86
Model 3	0.2 (-1.2, 1.6)	0.79
Model 4	-0.1 (-1.5, 1.3)	0.89
Mid-awakening (per 1day/week increase)		
Model 1	1.5 (0.2, 2.9)	0.03
Model 2	1.1 (-0.3, 2.4)	0.14
Model 3	1.0 (-0.4, 2.4)	0.16
Model 4	0.6 (-0.8, 2.0)	0.41
Early morning awakening (per 1day/week increase)		
Model 1	2.6 (0.9, 4.3)	0.004
Model 2	2.1 (0.4, 3.9)	0.02
Model 3	2.4 (0.7, 4.1)	0.01
Model 4	2.2 (0.5, 3.9)	0.01
Deep sleep disorder (per 1day/week increase)		
Model 1	1.6 (0.1, 3.2)	0.04
Model 2	1.7 (0.2, 3.2)	0.03
Model 3	1.8 (0.3, 3.3)	0.02
Model 4	1.5 (-0.02, 3.0)	0.06

IPFD, intra-pancreatic fat deposition; CI, confidence interval; MET, metabolic equivalent of task.

Model 1: not adjusted.

Model 2: adjusted for age and sex.

Model 3: adjusted for age, sex, and diabetes status.

Model 4: adjusted for age, sex, diabetes status, and body mass index.

### 3.3 Relationship between lifestyle factors and IHLA

In addition to IPFD, we examined the associations of IHLA score with lifestyle factors (**Table 3**) and nutrient intakes (**Supporting Information Table S3**). After adjusting for age, sex, diabetes status, and BMI, Brinkman index, difficulty falling asleep, deep sleep disorder were significantly associated with high IHLA score (p=0.04, 0.03, 0.03, respectively). Fast eating speed, late-night eating, and early morning awakening, which were significantly associated with IPFD score, were not associated with IHLA score. For nutrient intakes, while negative associations were observed between intakes of potassium, β-carotene, vitamin B6, vitamin C, and folate and IHLA in the univariate model (p=0.02, 0.03, 0.01, 0.04, 0.02, respectively), all these associations were attenuated after adjusted for age and sex.

**Table 3:** Relationship between lifestyle factors and IHLA score.

Eating habits		
Two meals/day (Yes/No)	Coefficient (95% CI)	p-value
Model 1	3.5 (-6.8, 13.8)	0.51
Model 2	-0.2 (-10.4, 10.1)	0.98
Model 3	3.1 (-6.8, 13.0)	0.54
Model 4	0.6 (-9.1, 10.3)	0.91
Number of snacks (per once/day increase)		
Model 1	0.1 (-4.7, 4.9)	0.96
Model 2	3.2 (-1.7, 8.1)	0.21
Model 3	1.6 (-3.2, 6.4)	0.50
Model 4	1.0 (-3.6, 5.6)	0.66
Fast eating (Yes/No)		
Model 1	5.1 (-2.4, 12.5)	0.19
Model 2	3.2 (-4.1, 10.5)	0.40
Model 3	0.8 (-6.3, 7.9)	0.83
Model 4	1.3 (-5.5, 8.1)	0.71
Late-night eating after 9:00 p.m. (Yes/No)		
Model 1	9.8 (-0.7, 20.3)	0.07
Model 2	5.3 (-5.4, 15.9)	0.34
Model 3	5.6 (-4.5, 15.7)	0.28
Model 4	4.9 (-4.8, 14.6)	0.33
Alcohol intake (per 1g/day increase)		
Model 1	0.03 (-0.2, 0.2)	0.77
Model 2	-0.02 (-0.2, 0.2)	0.87
Model 3	0.05 (-0.1, 0.2)	0.64
Model 4	0.1 (-0.1, 0.3)	0.42
Exercise habits		
Physical activity (per 1MET-hours/week increase)		
Model 1	-0.05 (-0.1, 0.04)	0.27
Model 2	-0.1 (-0.2, 0.01)	0.07
Model 3	-0.1 (-0.1, 0.03)	0.22
Model 4	-0.1 (-0.1, 0.02)	0.14
Smoking		
Brinkman index (per 1 increase)		
Model 1	0.01 (0.002, 0.02)	0.01
Model 2	0.01 (0.002, 0.02)	0.02
Model 3	0.01 (0.001, 0.02)	0.03
Model 4	0.01 (0.001,0.01)	0.04
Sleeping habits		
Short sleep duration (Yes/No)		
Model 1	4.5 (-4.6, 13.7)	0.59
Model 2	3.9 (-4.9, 12.6)	0.39
Model 3	6.0 (-2.3, 19.7)	0.16
Model 4	3.9 (-4.4, 18.9)	0.36
Difficulty falling asleep (per 1day/week increase)		
Model 1	1.8 (-.3, 3.3)	0.02
Model 2	1.7 (0.3, 3.2)	0.02
Model 3	1.8 (0.5, 3.2)	0.01
Model 4	1.5 (0.2, 2.9)	0.03
Mid-awakening (per 1day/week increase)		
Model 1	0.05 (-1.4, 1.5)	0.95
Model 2	0.4 (-1.1, 1.8)	0.62
Model 3	0.2 (-1.1, 1.6)	0.72
Model 4	-0.3 (-1.7, 1.0)	0.62
Early morning awakening (per 1day/week increase)		
Model 1	-1.1 (-2.9, 0.8)	0.26
Model 2	-0.5 (-2.4, 1.3)	0.56
Model 3	-0.2 (-1.9, 1.6)	0.83
Model 4	-0.5 (-2.2, 1.2)	0.57
Deep sleep disorder (per 1day/week increase)		
Model 1	2.0 (0.4, 3.6)	0.01
Model 2	1.8 (0.3, 3.3)	0.02
Model 3	2.1 (0.6, 3.5)	0.01
Model 4	1.6 (0.2, 3.1)	0.03

IHLA, intra-hepatic lipid accumulation. CI, confidence interval. MET, metabolic equivalent of task.

Model 1: not adjusted.

Model 2: adjusted for age and sex.

Model 3: adjusted for age, sex, and diabetes status.

Model 4: adjusted for age, sex, diabetes status, and body mass index.

### 3.4 Sensitivity analysis

Considering the possibility that IPFD changes depending on the presence or absence of diabetes, analysis was performed separately in the patients with and without diabetes (**Table 4**). In the patients with diabetes, fast eating speed were significantly associated with IPFD score (p<0.001), after adjusting for age, sex, and BMI. In the patients without diabetes, number of snacks, late-night eating and short sleep duration were significantly associated with IPFD score (p=0.02, 0.002 and 0.003, respectively), after adjusting for age, sex, and BMI.

Table 4Relationship between lifestyle factors and IPFD score (with/without diabetes).

**Table:** 

Eating habits	with diabetes (n = 68)		without diabetes (n = 31)	
Two meals/day (Yes/No)	Coefficient (95% CI)	p-value	Coefficient (95% CI)	p-value
Model 1	-6.6 (-20.2, 7.0)	0.35	14.0 (-0.9, 29.0)	0.08
Model 2	-6.8 (-21.0, 7.5)	0.35	17.0 (3.0, 30.9)	0.02
Model 3	-6.8 (-21.1, 7.6)	0.36	10.8 (-2.9, 24.5)	0.13
Number of snacks (per once/day increase)				
Model 1	-3.6 (-8.4, 1.2)	0.15	11.4 (2.0, 20.9)	0.02
Model 2	-3.9 (-9.3, 1.5)	0.16	11.6 (2.1, 21.0)	0.02
Model 3	-3.9 (-9.4, 1.5)	0.16	10.1 (1.9, 18.3)	0.02
Fast eating (Yes/No)				
Model 1	13.1 (5.7, 20.4)	<0.001	-10.8 (-24.6, 3.1)	0.14
Model 2	14.0 (6.4, 21.7)	<0.001	-7.6 (-21.3, 6.1)	0.29
Model 3	14.4 (6.6, 22.1)	<0.001	-9.6 (-21.2, 2.1)	0.12
Late-night eating after 9:00 p.m. (Yes/No)				
Model 1	6.5 (-5.5, 18.4)	0.29	15.6 (-1.6, 32.8)	0.09
Model 2	6.9 (-5.8, 19.6)	0.29	23.0 (7.3, 38.6)	0.01
Model 3	6.9 (-5.9, 19.7)	0.29	22.3 (9.3, 35.3)	0.002
Alcohol intake (per 1g/day increase)				
Model 1	-0.2 (-0.4, 0.1)	0.25	-0.2 (-0.5, 0.1)	0.27
Model 2	-0.2 (-0.5, 0.1)	0.25	-0.2 (-0.5, 0.1)	0.12
Model 3	-0.2 (-0.5, 0.1)	0.24	-0.2(-0.5, 0.05)	0.12

**Table:** 

Exercise habits				
Physical activity (per 1MET-hours/week increase)				
Model 1	0.01 (-0.1, 0.2)	0.90	0.03 (-0.1, 0.1)	0.49
Model 2	0.01 (-0.1, 0.2)	0.93	0.1 (-0.03, 0.2)	0.18
Model 3	0.01 (-0.1, 0.2)	0.93	0.05 (-0.1, 0.1)	0.34

**Table:** 

Smoking				
Brinkman index (per 1 increase)				
Model 1	0.003 (-0.005, 0.01)	0.48	0.03 (-0.01, 0.1)	0.16
Model 2	0.002 (-0.01, 0.01)	0.57	0.03 (-0.01, 0.1)	0.13
Model 3	0.002 (-0.01, 0.01)	0.58	0.01 (-0.03, 0.04)	0.75

**Table:** 

Sleeping habits				
Short sleep duration (Yes/No)				
Model 1	1.7 (-8.5, 12.0)	0.74	20.7 (6.3, 35.0)	0.01
Model 2	1.7 (-8.9, 12.2)	0.76	27.4 (15.3, 39.6)	<0.001
Model 3	1.7 (-8.9, 12.4)	0.75	22.0 (8.9, 35.1)	0.003
Difficulty falling asleep (days/week)				
Model 1	0.3 (-1.4, 1.9)	0.75	0.02 (-2.8, 2.9)	0.99
Model 2	0.3 (-1.4, 2.0)	0.75	0.4 (-2.3, 3.1)	0.80
Model 3	0.3 (-1.4, 2.0)	0.76	-1.7 (-4.3, 0.8)	0.20
Mid-awakening (days/week)				
Model 1	0.8 (-0.8, 2.3)	0.34	2.0 (-0.5, 4.5)	0.13
Model 2	0.8 (-0.9, 2.4)	0.36	1.5 (-1.0, 4.0)	0.24
Model 3	0.8 (-0.9, 2.4)	0.35	-0.3 (-2.8, 2.3)	0.84
Early morning awakening (days/week)				
Model 1	1.9 (0.02, 3.8)	0.05	4.0 (0.8, 7.1)	0.02
Model 2	2.0 (0.01, 3.9)	0.05	3.0 (-0.6, 6.5)	0.11
Model 3	2.0 (-0.0003, 3.9)	0.05	1.1 (-2.3, 4.6)	0.52
Deep sleep disorder (days/week)				
Model 1	1.4 (-0.4, 3.3)	0.13	2.3 (-0.2, 4.9)	0.09
Model 2	1.4 (-0.5, 3.3)	0.15	2.7 (0.3, 5.1)	0.04
Model 3	1.4 (-0.5, 3.3)	0.15	1.0 (-1.7, 3.7)	0.05

IPFD, intra-pancreatic fat deposition; CI, confidence interval; MET, metabolic equivalent of task.

Model 1: not adjusted.

Model 2: adjusted for age and sex.

Model 3: adjusted for age, sex, and body mass index.

## 4 Discussion

In the current study, we elucidated that fast eating speed, late-night eating and early morning awakening were significantly associated only with IPFD, not with IHLA, independently of age, sex, diabetes status, and BMI. To our knowledge, this is the first study to report the associations of dietary and sleeping habits with IPFD.

Several mechanisms may account for the association between fast eating and IPFD. Eating speed has been associated with obesity (21), metabolic syndrome (22), and the risk of type 2 diabetes mellitus (14). Fast eating causes a smaller postprandial decrease in ghrelin levels and a smaller increase in glucagon-like peptide-1 levels than does slow eating (23, 24), which result in postprandial hyperglycemia (25). As IPFD may accumulate because of a paracrine action of pancreatic local insulin secretion (17, 26), postprandial hyperglycemia and local hyperinsulinemia may explain the association between fast eating and IPFD, independent of the effects of obesity and IHLA. In fact, when we examined the association separately in the patients with and without diabetes, fast eating was significantly associated with IPFD only in patients with diabetes, who were prone to postprandial hyperglycemia.

The habit of late-night eating was also significantly associated with high IPFD score. Late-night eating leads to an elevation in hunger upon waking and a reduction in serum leptin levels (27). It also diminishes energy expenditure and core body temperature during wakefulness (27). In addition, it modifies adipose tissue gene expression favoring increased lipid storage (27) and decreases the thermic effect of food (28). Late-night eating has been reported to increase postprandial blood glucose level to the evening meal and the subsequent breakfast (29), which may lead to increased IPFD.

We have previously reported that skipping meals, which causes postprandial hyperglycemia following refeeding (30), was associated with IPFD in the patients with type 2 diabetes mellitus (8). Although in this study we did not observe an association between meal skipping and diabetes status possibly because of the small case number (only six patients with type 2 diabetes mellitus consumed two meals), consistent with other studies, our results suggest that avoiding eating patterns that cause postprandial hyperglycemia and hyperinsulinemia is important in terms of lowering the risk of IPFD.

In this study, early morning awakening, deep sleep disorder, and short sleep duration were associated with high IPFD score after adjusting for age, sex, and diabetes status. With further adjustment for BMI, the association between early morning awakening and high IPFD score remained significant. Early morning awakening, deep sleep disorder, and short sleep duration are known to be a risk factor for obesity (31, 32) and type 2 diabetes mellitus (33). They are linked to elevated levels of growth hormone and ghrelin, as well as reduced levels of leptin (34). Elevated growth hormone levels have been observed to hinder insulin receptors, causing insulin resistance (35). On the other hand, low levels of leptin and high levels of ghrelin are associated with an increased risk of obesity, either by reducing feelings of fullness or by stimulating appetite (36). Therefore, it is speculated that the association between IPFD and early morning awakening, deep sleep disorder, and short sleep duration may in part be explained by obesity. However, in the current study, the association between early morning awakening and IPFD remained significant after adjusting for BMI. A possible reason for this is that early morning awakening is a symptom of circadian rhythm sleep-wake disorders (37). The circadian rhythm is influenced by light exposure and diet, and it plays a crucial role in regulating metabolism (38). Circadian rhythm disturbances has been reported to be associated with elevated levels of both blood glucose and insulin (39), and this mechanism may explain the association between IPFD and circadian rhythm sleep-wake disorders.

For nutrient intakes, vitamin B6, folate, potassium, β-carotene, and vitamin C were negatively associated with IHLA in the univariate model, and vitamin B12 tended to be negatively associated with IPFD in the multivariate adjusted model. Vitamin B6, vitamin B12, and folate are involved in methionine metabolism, and their deficiency causes a decrease in phosphatidylcholine synthesis, which leads to accumulate ectopic fat (40). In addition, it has been reported that vitamin C and carotenoids may reduce fatty liver due to their antioxidant and anti-inflammatory effects (41). These mechanisms may explain the associations of nutrient intakes with IHLA and IPFD elucidated in this study.

There are several limitations of the present study that should be noted. First, this study used a cross-sectional design. The results can only show association and not causation. To demonstrate the causal relationship between lifestyle habits and IPFD, further longitudinal studies and intervention studies are needed. Also, residual confounding due to unmeasured variables or imprecise measurement of confounding should be considered. Second, the assessment of lifestyle is based on self-reported questionnaires. The results may not accurately reflect the patient’s actual lifestyle. Third, there was no histological confirmation of IPFD. However, the assessment method employed in this study has been validated in several prior studies, which demonstrated significant correlations between CT values and histologically determined fat quantities (16). Fourth, as the individuals included in this study were patients who were admitted to the hospital and received CT scans, generalizability may be limited. Fifth, the CT machines used in this study were not identical and scans obtained from imaging conditions with different tube voltages were included. However, this effect is thought to be minor because the CT values were adjusted by subtracting the spleen values (as a reference) for the assessment of ectopic fat.

In conclusion, the current study has elucidated the significant associations of fast eating speed, late-night eating, and early morning awakening with IPFD.

## Data availability statement

The raw data supporting the conclusions of this article will be made available by the authors, without undue reservation.

## Ethics statement

The studies involving human participants were reviewed and approved by Osaka University Hospital. The patients/participants provided their written informed consent to participate in this study.

## Author contributions

KM and MB conceived and designed the study and performed statistical analysis. KM, MB, SK, AN, HO, SM, CI, YH, YF, AT, TN, and JK interpreted the data. KM and MB drafted the manuscript. KM and MB are the guarantors of this work and had full access to all the data in the study and take responsibility for the integrity of the data and the accuracy of the data analysis. IS and JK critically revised the manuscript. All authors contributed to the article and approved the submitted version.
