# Supplementary material for: Relationships between intra-pancreatic fat deposition and lifestyle factors: a cross-sectional study
Source: Front Endocrinol (Lausanne). 2023 Jul 27;14:1219579. doi: 10.3389/fendo.2023.1219579 (PMC10415674; doi:10.3389/fendo.2023.1219579)
Supplement: Supplementary file 1 [file Table_1.docx]

**Supporting Information Table S1. Questionnaire we used in this study**

| Question item | Question content | Answer |
| --- | --- | --- |
| Brinkman Index | Have you ever smoked? If yes, how many years have you smoked? | Never smoked / Currently smokes ( cigarettes/day years) / Quit smoking ( cigarettes/day years) |
| Two meals/day Late-night eating | Please indicate the average time you eat. | 1st meal ：  2nd meal ：  3rd meal　： 4th meal　： |
| Fast eating | How fast do you eat? | Very fast / fast / mid / slow / very slow |
| Number of snacks | On average, how many snacks do you have during the day? | none / once / twice / three times or more |
| Sleep duration | How many hours do you usually sleep? | 5 hours or less / 6 hours / 7 hours / 8 hours / 9 hours / 10 hours or more |
| Difficulty falling asleep | How many times a week did you go to bed and not fall asleep within 30 minutes? | Rarely / Less than once a week / 1-2 times a week / 3-4 times a week / 5-6 times a week / Almost every day |
| Mid-awakening | How many times a week did you wake up in the middle of the night or early in the morning? | Rarely / Less than once a week / 1-2 times a week / 3-4 times a week / 5-6 times a week / Almost every day |
| Early morning awakening | How many times a week did you wake up early in the morning and not be able to go back to sleep? | Rarely / Less than once a week / 1-2 times a week / 3-4 times a week / 5-6 times a week / Almost every day |
| Deep sleep disorder | How many times a week have you had a light sleep and not felt like you slept well? | Rarely / Less than once a week / 1-2 times a week / 3-4 times a week / 5-6 times a week / Almost every day |

**Supporting Information Table S2. Relationship between nutrient intakes and IPFD score**

| Carbohydrates (per 1g increase) | Coefficient (95% CI) | p-value |
| --- | --- | --- |
| Model 1 | -0.01 (-0.2, 0.1) | 0.89 |
| Model 2 | 0.02 (-0.1, 0.2) | 0.82 |
| Model 3 | 0.01 (-0.1, 0.2) | 0.94 |
| Model 4 | 0.01 (-0.1, 0.2) | 0.94 |
| Protein (per 1g increase) |  |  |
| Model 1 | 0.3 (-0.4, 0.9) | 0.47 |
| Model 2 | 0.03 (-0.7, 0.7) | 0.94 |
| Model 3 | -0.1 (-0.8, 0.6) | 0.87 |
| Model 4 | -0.1 (-0.7, 0.6) | 0.86 |
| Lipid (per 1g increase) |  |  |
| Model 1 | 0.4 (-0.5, 1.3) | 0.39 |
| Model 2 | 0.4 (-0.6, 1.3) | 0.44 |
| Model 3 | 0.3 (-0.7, 1.2) | 0.57 |
| Model 4 | 0.3 (-0.7, 1.2) | 0.55 |
| Saturated fatty acid (per 1g increase) |  |  |
| Model 1 | 0.9 (-1.1, 3.0) | 0.37 |
| Model 2 | 1.3 (-0.9, 3.6) | 0.25 |
| Model 3 | 1.0 (-1.3, 3.3) | 0.39 |
| Model 4 | 1.15 (-1.1, 3.4) | 0.31 |
| Dietary fiber (per 1g increase) |  |  |
| Model 1 | 1.0 (-0.9, 2.9) | 0.30 |
| Model 2 | 0.5 (-1.8, 2.7) | 0.69 |
| Model 3 | 0.4 (-1.8, 2.6) | 0.73 |
| Model 4 | 0.4 (-1.7, 2.6) | 0.69 |
| Potassium (per 1mg increase) |  |  |
| Model 1 | 0.01 (-0.002, 0.02) | 0.12 |
| Model 2 | 0.01 (-0.01, 0.02) | 0.26 |
| Model 3 | 0.01 (-0.01, 0.02) | 0.33 |
| Model 4 | 0.01 (-0.005, 0.02) | 0.25 |
| Calcium (per 1mg increase) |  |  |
| Model 1 | 0.03 (-0.004, 0.1) | 0.09 |
| Model 2 | 0.03 (-0.01, 0.1) | 0.13 |
| Model 3 | 0.02 (-0.01, 0.1) | 0.20 |
| Model 4 | 0.02 (-0.01, 0.1) | 0.14 |
| Iron (per 1mg increase) |  |  |
| Model 1 | 1.0 (-2.4, 4.5) | 0.57 |
| Model 2 | -0.2 (-3.9, 3.5) | 0.92 |
| Model 3 | -0.2 (-3.9, 3.4) | 0.90 |
| Model 4 | -0.3 (-3.9, 3.3) | 0.87 |
| β-carotene (per 1μg increase) |  |  |
| Model 1 | 0.001 (-0.002,0.005) | 0.38 |
| Model 2 | 0.002 (-0.002, 0.01) | 0.41 |
| Model 3 | 0.001 (-0.003, 0.005) | 0.54 |
| Model 4 | 0.002 (-0.002, 0.01) | 0.36 |
| Vitamin B6 (per 1mg increase) |  |  |
| Model 1 | 13.0 (-13.8, 39.7) | 0.35 |
| Model 2 | 2.1 (-26.5, 30.7) | 0.89 |
| Model 3 | 1.3 (-27.0, 29.6) | 0.93 |
| Model 4 | 1.7 (-26.0, 29.4) | 0.90 |
| Vitamin B12 (per 1μg increase) |  |  |
| Model 1 | -0.7 (-3.9, 2.4) | 0.64 |
| Model 2 | -3.4 (-7.1, 0.3) | 0.08 |
| Model 3 | -3.8 (-7.5, -0.1) | 0.05 |
| Model 4 | -3.6 (-7.2, 0.02) | 0.05 |
| Vitamin C (per 1mg increase) |  |  |
| Model 1 | 0.2 (-0.03, 0.4) | 0.11 |
| Model 2 | 0.2 (-0.1, 0.4) | 0.18 |
| Model 3 | 0.1 (-0.1, 0.3) | 0.25 |
| Model 4 | 0.2 (-0.1, 0.4) | 0.16 |
| Folate (per 1μg increase) |  |  |
| Model 1 | 0.03 (-0.03, 0.1) | 0.28 |
| Model 2 | 0.02 (-0.1, 0.1) | 0.61 |
| Model 3 | 0.01 (-0.1, 0.1) | 0.70 |
| Model 4 | 0.02 (-0.1, 0.1) | 0.62 |
| Salt (per 1g increase) |  |  |
| Model 1 | 2.2 (-0.5, 4.9) | 0.11 |
| Model 2 | 1.9 (-1.0, 4.8) | 0.20 |
| Model 3 | 1.6 (-1.3, 4.5) | 0.28 |
| Model 4 | 1.5 (-1.3, 4.4) | 0.29 |
| n-3 fatty acid (per 1g increase) |  |  |
| Model 1 | 3.8 (-15.0, 22.7) | 0.69 |
| Model 2 | -7.4 (-29.3, 14.6) | 0.51 |
| Model 3 | -7.4 (-29.1, 14.3) | 0.51 |
| Model 4 | -9.6 (-30.8, 11.7) | 0.38 |
| n-6 fatty acid (per 1g increase) |  |  |
| Model 1 | 1.2 (-3.6, 6.0) | 0.62 |
| Model 2 | 0.5 (-4.3, 5.3) | 0.83 |
| Model 3 | 0.6 (-4.1, 5.4) | 0.79 |
| Model 4 | -0.01 (-4.7, 4.7) | 1.00 |
| Isoflavone (per 1mg increase) |  |  |
| Model 1 | 0.04 (-0.1, 0.2) | 0.55 |
| Model 2 | 0.02 (-0.1, 0.2) | 0.81 |
| Model 3 | 0.02 (-0.1, 0.1) | 0.82 |
| Model 4 | 0.02 (-0.1, 0.1) | 0.80 |

IPFD, intra-pancreatic fat deposition. CI, confidence interval.

Model 1: not adjusted.

Model 2: adjusted for age and sex.

Model 3: adjusted for age, sex, and diabetes status.

Model 4: adjusted for age, sex, diabetes status, and body mass index.

**Supporting Information Table S3. Relationship between nutrient intakes and IHLA score**

| Carbohydrates (per 1g increase) | Coefficient (95% CI) | p-value |
| --- | --- | --- |
| Model 1 | 0.1 (-0.05, 0.3) | 0.17 |
| Model 2 | 0.1 (-0.02, 0.3) | 0.09 |
| Model 3 | 0.1 (-0.03, 0.3) | 0.13 |
| Model 4 | 0.1 (-0.02, 0.2) | 0.11 |
| Protein (per 1g increase) |  |  |
| Model 1 | -0.4 (-1.1, 0.3) | 0.32 |
| Model 2 | -0.2 (-0.9, 0.5) | 0.56 |
| Model 3 | -0.4 (-1.0, 0.3) | 0.29 |
| Model 4 | -0.4 (-1.0, 0.3) | 0.26 |
| Lipid (per 1g increase) |  |  |
| Model 1 | -0.9 (-1.8, 0.01) | 0.06 |
| Model 2 | -0.5 (-1.5, 0.4) | 0.30 |
| Model 3 | -0.7 (-1.6, 0.2) | 0.13 |
| Model 4 | -0.7 (-1.6, 0.2) | 0.12 |
| Saturated fatty acid (per 1g increase) |  |  |
| Model 1 | -2.0 (-4.0, 0.1) | 0.06 |
| Model 2 | -0.9 (-3.2, 1.3) | 0.41 |
| Model 3 | -1.6 (-3.8, 0.5) | 0.14 |
| Model 4 | -1.5 (-3.5, 0.6) | 0.17 |
| Dietary fiber (per 1g increase) |  |  |
| Model 1 | -1.9 (-3.8, 0.04) | 0.06 |
| Model 2 | -0.3 (-2.5, 1.9) | 0.79 |
| Model 3 | -0.4 (-2.5, 1.7) | 0.70 |
| Model 4 | -0.4 (-2.4, 1.7) | 0.73 |
| Potassium (per 1mg increase) |  |  |
| Model 1 | -0.01 (-0.02, -0.002) | 0.02 |
| Model 2 | -0.01 (-0.02, 0.01) | 0.37 |
| Model 3 | -0.01 (-0.02, 0.004) | 0.21 |
| Model 4 | -0.01 (-0.02, 0.005) | 0.27 |
| Calcium (per 1mg increase) |  |  |
| Model 1 | -0.02 (-0.05, 0.01) | 0.26 |
| Model 2 | -0.002 (-0.04, 0.03) | 0.91 |
| Model 3 | -0.01 (-0.04, 0.02) | 0.55 |
| Model 4 | -0.01 (-0.04, 0.02) | 0.65 |
| Iron (per 1mg increase) |  |  |
| Model 1 | -3.1 (-6.6, 0.4) | 0.09 |
| Model 2 | -1.1 (-4.8, 2.6) | 0.58 |
| Model 3 | -1.1 (-4.7, 2.4) | 0.53 |
| Model 4 | -1.3 (-4.6, 2.1) | 0.47 |
| β-carotene (per 1μg increase) |  |  |
| Model 1 | -0.004 (-0.01, 0.0005) | 0.03 |
| Model 2 | -0.001 (-0.005, 0.002) | 0.43 |
| Model 3 | -0.002 (-0.01, 0.001) | 0.20 |
| Model 4 | -0.002 (-0.005, 0.002) | 0.34 |
| Vitamin B6 (per 1mg increase) |  |  |
| Model 1 | -34.1 (-60.6, -7.6) | 0.01 |
| Model 2 | -21.1 (-49.3, 7.1) | 0.15 |
| Model 3 | -22.4 (-49.2, 4.4) | 0.10 |
| Model 4 | -21.9 (-47.5, 3.7) | 0.10 |
| Vitamin B12 (per 1μg increase) |  |  |
| Model 1 | -0.2 (-3.4, 3.0) | 0.92 |
| Model 2 | -1.6 (-5.4, 2.1) | 0.40 |
| Model 3 | -2.2 (-5.8, 1.3) | 0.22 |
| Model 4 | -2.0 (-5.4, 1.5) | 0.26 |
| Vitamin C (per 1mg increase) |  |  |
| Model 1 | -0.2 (-0.4, 0.01) | 0.04 |
| Model 2 | -0.1 (-0.3, 0.1) | 0.45 |
| Model 3 | -0.1 (-0.3, 0.1) | 0.24 |
| Model 4 | -0.1 (-0.3, 0.1) | 0.34 |
| Folate (per 1μg increase) |  |  |
| Model 1 | -0.1 (-0.1, -0.01) | 0.02 |
| Model 2 | -0.03 (-0.1, 0.04) | 0.38 |
| Model 3 | -0.04 (-0.1, 0.03) | 0.25 |
| Model 4 | -0.04 (-0.1, 0.03) | 0.28 |
| Salt (per 1g increase) |  |  |
| Model 1 | -1.9 (-4.7, 0.8) | 0.17 |
| Model 2 | -0.2 (-3.1, 2.7) | 0.88 |
| Model 3 | -0.8 (-3.6, 2.0) | 0.56 |
| Model 4 | -0.9 (-3.5, 1.8) | 0.51 |
| n-3 fatty acid (per 1g increase) |  |  |
| Model 1 | -4.1 (-23.3, 15.0) | 0.67 |
| Model 2 | -12.1 (-33.9, 9.7) | 0.28 |
| Model 3 | -12.2 (-32.9, 8.6) | 0.25 |
| Model 4 | -15.0 (-34.8, 4.8) | 0.14 |
| n-6 fatty acid (per 1g increase) |  |  |
| Model 1 | -2.9 (-7.8, 1.9) | 0.23 |
| Model 2 | -1.2 (-6.0, 3.6) | 0.63 |
| Model 3 | -1.0 (-5.5, 3.6) | 0.68 |
| Model 4 | -1.9 (-6.2, 2.5) | 0.41 |
| Isoflavone (per 1mg increase) |  |  |
| Model 1 | -0.1 (-0.2, 0.1) | 0.39 |
| Model 2 | -0.01 (-0.1, 0.1) | 0.84 |
| Model 3 | -0.01 (-0.1, 0.1) | 0.82 |
| Model 4 | -0.01 (-0.1, 0.1) | 0.83 |

IHLA, intra-hepatic lipid accumulation. CI, confidence interval.

Model 1: not adjusted.

Model 2: adjusted for age and sex.

Model 3: adjusted for age, sex, and diabetes status.

Model 4: adjusted for age, sex, diabetes status, and body mass index.
