# Supplementary figures and images for: Relationships between intra-pancreatic fat deposition and lifestyle factors: a cross-sectional study
Source: Front Endocrinol (Lausanne). 2023 Jul 27;14:1219579. doi: 10.3389/fendo.2023.1219579 (PMC10415674; doi:10.3389/fendo.2023.1219579)

## Slide 1
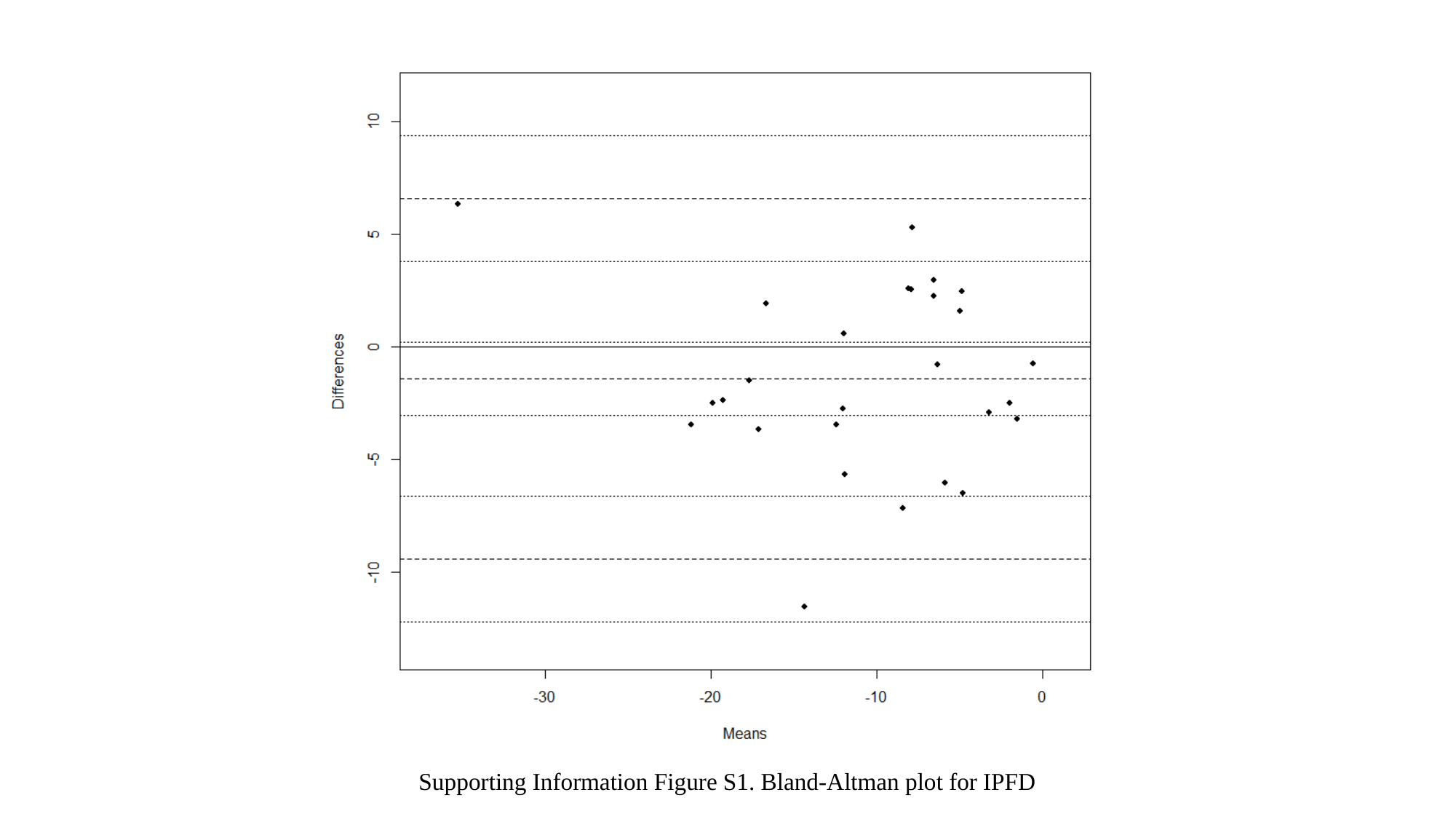

Supporting Information Figure S1. Bland-Altman plot for IPFD

Supplement: Supplementary file 2 [file Presentation_1.pptx]

## Slide 1
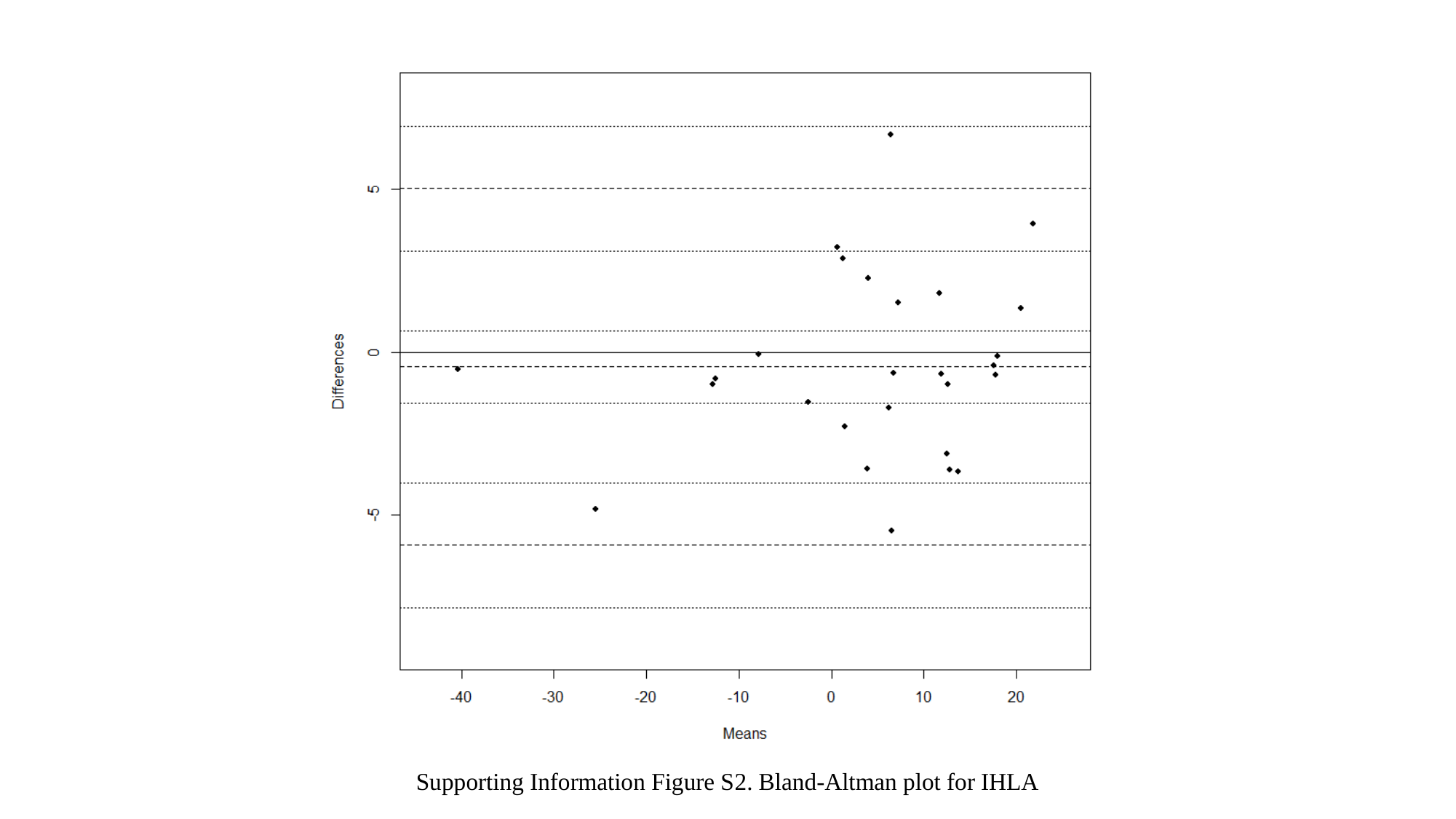

Supporting Information Figure S2. Bland-Altman plot for IHLA

Supplement: Supplementary file 3 [file Presentation_2.pptx]
